# miRNA-23a/CXCR4 regulates neuropathic pain via directly targeting TXNIP/NLRP3 inflammasome axis

**DOI:** 10.1186/s12974-018-1073-0

**Published:** 2018-01-31

**Authors:** Zhiqiang Pan, Qun Shan, Pan Gu, Xiao Min Wang, Lydia Wai Tai, Menglan Sun, Xin Luo, Liting Sun, Chi Wai Cheung

**Affiliations:** 1 Jiangsu Province Key Laboratory of Anesthesiology, Xuzhou Medical University, Xuzhou, 221002 China; 2 Laboratory and Clinical Research Institute for Pain, Department of Anesthesiology, The University of Hong Kong, Hong Kong SAR, China; 3 School of Life Science, Jiangsu Normal University, Xuzhou, 221116 Jiangsu Province People’s Republic of China; 4 Research Centre of Heart, Brain, Hormone and Healthy Aging, The University of Hong Kong, Hong Kong SAR, China; 5 Department of Anaesthesiology, Queen Mary Hospital, The University of Hong Kong, Rm 424, 4/F, Block K, 102 Pokfulam, Hong Kong, China

**Keywords:** miRNA-23a, CXCR4, TXNIP, NLRP3 inflammasome, Sciatic nerve injury, Spinal glia cell

## Abstract

**Background:**

Chemokine CXC receptor 4 (CXCR4) in spinal glial cells has been implicated in neuropathic pain. However, the regulatory cascades of CXCR4 in neuropathic pain remain elusive. Here, we investigated the functional regulatory role of miRNAs in the pain process and its interplay with CXCR4 and its downstream signaling.

**Methods:**

miRNAs and CXCR4 and its downstream signaling molecules were measured in the spinal cords of mice with sciatic nerve injury via partial sciatic nerve ligation (pSNL). Immunoblotting, immunofluorescence, immunoprecipitation, and mammal two-hybrid and behavioral tests were used to explore the downstream CXCR4-dependent signaling pathway.

**Results:**

CXCR4 expression increased in spinal glial cells of mice with pSNL-induced neuropathic pain. Blocking CXCR4 alleviated the pain behavior; contrarily, overexpressing CXCR4 induced pain hypersensitivity. MicroRNA-23a-3p (miR-23a) directly bounds to 3′ UTR of CXCR4 mRNA. pSNL-induced neuropathic pain significantly reduced mRNA expression of miR-23a. Overexpression of miR-23a by intrathecal injection of miR-23a mimics or lentivirus reduced spinal CXCR4 and prevented pSNL-induced neuropathic pain. In contrast, knockdown of miR-23a by intrathecal injection of miR-23a inhibitor or lentivirus induced pain-like behavior, which was reduced by CXCR4 inhibition. Additionally, miR-23a knockdown or CXCR4 overexpression in naïve mice could increase the thioredoxin-interacting protein (TXNIP), which was associated with induction of NOD-like receptor protein 3 (NLRP3) inflammasome. Indeed, CXCR4 and TXNIP were co-expressed. The mammal two-hybrid assay revealed the direct interaction between CXCR4 and TXNIP, which was increased in the spinal cord of pSNL mice. In particular, inhibition of TXNIP reversed pain behavior elicited by pSNL, miR-23a knockdown, or CXCR4 overexpression. Moreover, miR-23a overexpression or CXCR4 knockdown inhibited the increase of TXNIP and NLRP3 inflammasome in pSNL mice.

**Conclusions:**

miR-23a, by directly targeting CXCR4, regulates neuropathic pain via TXNIP/NLRP3 inflammasome axis in spinal glial cells. Epigenetic interventions against miR-23a, CXCR4, or TXNIP may potentially serve as novel therapeutic avenues in treating peripheral nerve injury-induced nociceptive hypersensitivity.

**Electronic supplementary material:**

The online version of this article (10.1186/s12974-018-1073-0) contains supplementary material, which is available to authorized users.

## Background

Chemokine CXC receptor 4 (CXCR4) belongs to the family of G protein-coupled receptors. CXCR4 has been confirmed to have glia-modulatory and neuromodulatory properties in the central nervous system (CNS) [1]. Mounting evidence has shown that CXCR4 is involved in the process of different nociceptive responses such as neuropathic pain or cancer pain in glial cells of the dorsal root ganglion (DRG) or in the spinal cord [2–4]. Recently, it has been found that both chemokine C-X-C motif ligand 12 (CXCL12) and its receptor CXCR4 were upregulated in spinal glial cells of mice with partial sciatic nerve ligation (pSNL)-induced neuropathic pain or chronic post-ischemia-induced inflammatory pain [5]. Inhibition of CXCR4 attenuated pain induced by CXCL12, suggesting that the crosstalk between astrocytic CXCL12 and microglial CXCR4 contributes to the development of neuropathic pain [5]. However, the functional regulatory mechanisms of spinal CXCR4 in neuropathic pain remain unclear.

Recent reports have strongly linked miRNA to nociceptive processing. MicroRNA-23a-3p (miR-23a) is highly conserved across species, and it modulates various disease processes, such as cancer [6], inflammation [7], Harada Miuji syndrome [8], and cognitive impairment [9]. Moreover, miR-23a was found not only decreased in the blood of patients with multiple sclerosis [10] or acute ischemic stroke [11], but also rapidly downregulated in the injured cortex following traumatic brain injury [12], suggesting a potential modulatory function of miR-23a in CNS diseases. Here, miR-23a was predicted to bind to CXCR4 mRNA; however, it is still unknown whether miR-23a regulated neuropathic pain via directly targeting CXCR4.

Thioredoxin-interacting protein (TXNIP) is ubiquitously expressed in a variety of cells and acts as an endogenous suppressor of reactive oxygen species scavenging protein thioredoxin, as well as a crucial molecular nutrient sensor to oxidative stress and inflammation in the regulation of energy metabolism [13, 14]. TXNIP is associated with stroke, depression, Alzheimer’s disease, and spinal or brain injury [15–18]. Knockdown of hippocampal TXNIP significantly improves brain injury [15], cognitive impairment, and neuroinflammation [16], suggesting that TXNIP is a potential target for the treatment of these CNS disorders. In particular, overexpression of TXNIP attenuates CXCL12-induced bladder carcinogenesis, while knockout of TXNIP enhances CXCR4 expression in bladder carcinogenesis in urothelial cells [19], indicating an interaction may exist between TXNIP and CXCR4. Therefore, the present study aimed to determine the functional and regulatory role of miR-23a in pain processing in the CNS and its interplay with CXCR4 and TXNIP at spinal level, which may provide potential therapeutic targets for peripheral injury-induced neuropathic pain.

## Methods

### Animals

Pathogen-free adult male C57BL/6J wild-type mice (25–30 g, *n* = 3 or 5 per group for each experiment) were housed at 23 ± 3 °C, with humidity ranges between 25 and 45% under a 12-h light/12-h dark cycle (lights on at 07:00). Mice were allowed for free access to water and standard lab diet (1.0% calcium, 0.5% phosphorus, and 3.3 IU/g of vitamin D3).

### pSNL-induced neuropathic pain model

The pSNL model is an animal model of peripheral neuropathic pain as described [20]. Mice were anesthetized with inhalation anesthesia by isoflurane in O_2_. Under an anesthesia condition, the right sciatic nerve was exposed by an incision from the right sciatic notch to the distal thigh. By using the femoral head as a landmark, the location of the sciatic nerve ligation was identified. Approximately half of the sciatic nerve was tightly ligated with a 7-0 silk suture. The incision was closed with a 5-0 cotton suture and disinfected with ethanol. For the sham operation, the right sciatic nerve was only exposed but not ligated.

### Behavioral assessment

Behavior tests were conducted, with the experimenter blind of group assignment. Paw withdrawal thresholds (PWTs) to mechanical stimulus (a measure of allodynia) and paw withdrawal latency (PWL) to thermal stimulus (a measure of hyperalgesia) were performed as described previously [21]. Briefly, mice were placed individually in plexiglass boxes on a stainless-steel mesh floor for 30 min to acclimate the environment. Hind PWT, which responses to blunted von Frey filaments connected to a calibrated electronic von Frey filament esthesiometer (IITC Life Science Inc., Woodland Hill, CA, USA), was recorded automatically by the esthesiometer. Proper paw withdrawal threshold was elicited by a range of probes (1–10 g). The weakest filament that was able to elicit a paw withdrawal response to this filament defined if a series of weaker or stronger filaments would be tested [22]. The filaments were applied perpendicularly to the plantar surface of the ipsilateral hind paw (surgery side). Quick withdrawal (but not due to locomotion) or licking of the paw was considered as a positive response. Five measurements were made per animal per test session with intervals of 3 min, and the mean value of these five repetitions was counted as the mechanical threshold of a hind paw. Only animals that displayed mechanical hypersensitivity (at least 30% reduction of PWT relative to baseline) to von Frey probes after pSNL were included. The degree of thermal hyperalgesia was measured using Hargreaves’ test (Ugo Basile, Varese, Italy). Before testing, mice were individually placed in a plexiglass chamber on a transparent glass platform. After 30 min of acclimation, an infrared beam was directed toward the same area of the hind paw, as described for von Frey assay, and PWL was recorded. A 20-s cutoff was used to prevent tissue damage. The light beam intensity was adjusted by radiometer before and after every test session. Five measurements were made per animal in each test section with intervals of at least 5 min.

### Drug application

Drugs were delivered intrathecally by a direct lumbar puncture method in awake mice as described previously [23]. In brief, lumbar puncture was performed by gently gripping the iliac crest of the mouse and inserting a 50-μl Hamilton microsyringe with a 30-gauge needle into the subdural space of the spinal L3–L5 levels. Successful puncture was indicated by a tail flick.

### RNA, miRNA isolation, and qRT-PCR

Ipsilateral dorsal quadrants of lumbar spinal cord segments (L3–L5) were homogenized in Trizol (15596-026, Invitrogen). Reverse transcription of miRNA was performed using the primer 23RT (5′-TTAACTGGATACGAAGGGTCCGAACACCGGTCGTATCCAGT TAAAGGAAATC-3′). Transcription of other genes was performed with oligo (dT)_18_. cDNA products were used as templates to detect miRNA or gene content in quantitative reverse transcription PCR (qRT-PCR), respectively, using primers 23 (5′-TGCGTGCGA TCACATTGCCAGGGA-3′ and 5′-TACGAAGGGTCCGAACAC-3′) and TXNIP (5′-T CTTTTGAGGTGGTCTTCAACG-3′ and 5′-GCTTTGACTCGGGTAACTTCACA-3′). SYBR Green method was used to perform qRT-PCR with the SYBR PremixEx TaqII kit (RR820A, Takara). Each reaction was run in triplicate. Ct method was used to analyze the expression as previously described (Pan et al. [24]). U6 (5′-CTCGCTTCGGCAGCAC ATATACT-3′ and 5′-ACGCTTCACGAATTTGCGTGTC-3′) was used as an internal control for miRNA; Gapdh (5′-GGTGAAGGTCGGTGTGAACG-3′ and 5′-CTCGCTC CTGGAAGATGGTG-3′) was used as an internal control.

### Plasmid construction

To construct expression vectors, oligos were synthesized in specific sequences listed as follows: miR-23a overexpression primer 23W (5′-AGCTCGAGAGACCCAGCCTGGT CAAGAT-3′ and 5′-GTACGCGTTCATGATAGGCTTCTCTGTTA-3′), CXCR4 overexpression primer C4W (5′-AGCTCGAGATGGAACCGATCAGTGTGA-3′ and 5′- GTACGCGTGTGTTAGCTGGAGTGAAAAC-3′), and miR-23a knockdown primer LV-23 (5′-PCGCGGGAAATCCCAACCAATGTGATGCTAGGAAATCCCAACCAAT GTGAT-3′ and 5′-P-CGCCTTTAGGGTTGGTTACACTACGATCCTTTAGGGTTGGT TACACTA-3′) and then amplified in PCR. To construct vectors for gene overexpression, purified PCR products were digested with the PWPXL vector together using Xhol and MluI then ligated with T4 ligase. For miR-23a knockdown construct, pLVTHM vector was digested with ClaI and MluI and then ligated to annealed double-strand oligos LV-23 using T4 ligase. All constructs were confirmed by DNA sequencing.

### Lentivirus package and infection

**Fig. 1:**
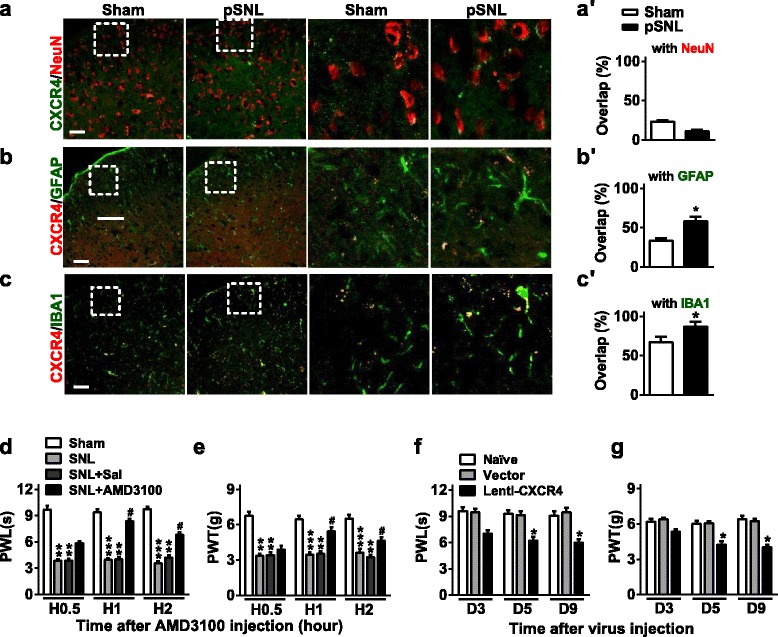
CXCR4 modulates neuropathic pain. **a**–**c** CXCR4 immunofluorescent co-staining with NeuN (a neuron marker) (**a**, **a′**), GFAP (an astrocyte marker) (**b**, **b′**) or IBA1 (a microglial marker) (**c**, **c′**) in the lumbar segment of the spinal cord at 7 days after pSNL surgery. *p < 0.05, versus Sham group by two-tailed unpaired Student’s *t* test; *n* = 5 per group. Original magnification × 200 for all the confocal images. Scale bar, 50 μm. **d**, **e** The effect of intrathecal injection of CXCR4 antagonist AMD3100 on the pSNL-induced thermal hyperalgesia (PWL) (**d**) and mechanical allodynia (PWT) (**e**) on day 7 after pSNL. Behavior was tested at 0.5, 1, and 2 h after AMD3100 injection. One-way ANOVA (behavior change versus treatment groups) followed by post hoc Tukey test. PWL F (6, 48) = 5.624; PWT F (6, 48) = 4.071, **p < 0.01, ***p < 0.001 versus Sham group; ^#^*p* < 0.05 versus pSNL+Sal group. **f**, **g** Daily intrathecal injections of Lenti-CXCR4 for 3 consecutive days in naïve mice induced thermal hyperalgesia (**f**) and mechanical allodynia (**g**). Behavior was tested 48 h after lentivirus injection in naïve mice. One-way ANOVA (behavior change versus treatment groups) followed by post hoc Tukey test. PWL F (4, 36) = 57.264; PWT F (4, 36) = 3.194, *p < 0.05, versus vector group. Data are presented as mean ± SEM; *n* = 5 per group

The constructed core plasmid (16 μg) and two envelope plasmids, PSPAX2 (12 μg) and PMD2G (4.8 μg), were co-transfected into HEK293T cells in a 6-well plate according to manufacturer instructions of Lipofectamine 2000 (11668-027, Invitrogen). The supernatant was collected at 48 h after transfection and concentrated by using a Centricon Plus-70 filter unit (UFC910096, Millipore). Lentivirus with titers 10^8^ TU/ml was used in the experiment. The assays of lentivirus in vitro and in vivo infection were performed according to a previous study [24]. Briefly, 20 μl lentivirus and 1.5 μl polybrene (1.4 μg/μl; H9268, Sigma-Aldrich) were added in a 24-well plate containing 1 × 10^5^ HEK293T cells and DMEM, without FBS; after 24 h, the transfection medium was replaced with 500 μl fresh complete medium containing 10% FBS; cells were collected at 48 h after culture. In general, daily intrathecal injections of lentivirus or vector (1 μl) were performed for 3 consecutive days in naïve or pSNL (beginning 7 days after pSNL) mice before sample collection or further behavior tests to allow sufficient overexpression or knockdown. Otherwise, please see specified injection time points detailed in corresponding figure legend of Fig. 1.

### siRNA, mimics, inhibitor, and antagonist delivery

**Fig. 2:**
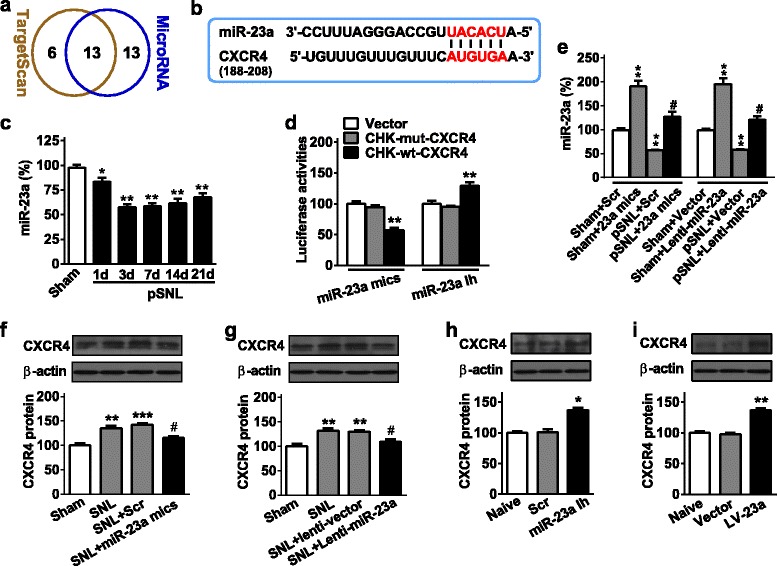
miR-23a regulates CXCR4 expression in spinal astrocyte in neuropathic pain. **a** The shared miRNA numbers to targeting CXCR4 predicted by the use of TargetScan and MicroRNA program. **b** The informatics analysis of miR-23a binding the 3′UTR in CXCR4 mRNA. **c** Time course of spinal miR-23a expression in pSNL-induced chronic neuropathic pain mice. One-way ANOVA (expression versus time point) followed by post hoc Tukey test, *F*_time_ (5, 24) = 19.66, *p < 0.05, **p < 0.01 versus sham group. **d** In vitro validation of miR-23a targeting CXCR4. A mutation was generated in the CXCR4-3′-UTR mRNA sequence in the complementary site for the seed region of miR-23a as indicated (CHK-mut-CXCR4). **p < 0.01 versus the corresponding CHK-mut-CXCR4 or empty vector group by two-tailed unpaired Student’s *t* test. **e** The validation of transfection efficiency of miR-23 mimics or Lenti-miR-23a in the mouse spinal cord by qRT-PCR. Spinal cord was harvested 24 h after intrathecal injection of continuous 2-day miR-23a mimics in naïve mice or pSNL mice with 7-day surgery or 72 h after intrathecal injection of continuous 2-day Lenti-miR-23a in naïve mice or pSNL mice with 7-day surgery. **f**, **g** The increased spinal CXCR4 protein expression in pSNL mice was reversed by intrathecal injection of miR-23a mimics (**f**) or Lenti-miR-23a (**g**). Intrathecal injections of miR-23a mimics or Lenti-miR-23a were performed from day 7 after pSNL. CXCR4 was measured at 24 h after 2-day miR-23a mimics injections or 48 h after 3-day Lenti-miR-23a injections. One-way ANOVA (expression versus treatment groups) followed by post hoc Tukey test, **f** *F* (3, 16) = 11.2, **g** *F*_time_ (3, 16) = 14.6, **p < 0.01, ***p < 0.001, sham group; ^#^*p* < 0.05 versus pSNL+Scr or pSNL+Lenti-vector group. **h**, **i** Spinal CXCR4 protein expression was increased by intrathecal injection of miR-23a inhibitor (miR-23a Ih) (**h**) or LV-miR-23a (LV-23a) (**i**) in naïve mice. CXCR4 was measured at 24 h after 2-day miR-23a inhibitor injections or at 48 h after 3-day LV-miR-23a injections. One-way ANOVA (expression versus treatment groups) followed by post hoc Tukey test, **h** *G* (2, 12) = 26.98, **i** *H* (2, 12) = 24.56, *p < 0.05, **p < 0.01 versus Scr or vector group. Data are presented as mean ± SEM; *n* = 5 per group

TXNIP siRNAs (681si, 5′-CCAGCCAACUCAAGAGGCAAAGAAAUU-3′, and 5′-U UUCUUUGCCUCUUGAGUUGGCUGGUU-3′; 1271si, 5′-GCCUCAGAGUGCAGA AGAUUUUU-3′, and 5′-AAAUCUUCUGCACUCUGAGGCUU-3′), miR-23a mimics (5′-UAAUGCCCCUAAAAAUCCUUAU-3′ and 5′-AUAAGGAUUUUUAGGGGCAU UA-3′), miR-23a inhibitor (Ih) (5′-AUAAGGAUUUUUAGGGGCAUUA-3′), CXCR4 smart pool siRNAs (94si and 5′-GAACCGAUCAGUGUGAGUA-3′, 192si and 5′-AAACGUC CAUUUCAAUAGG-3′, 420si and 5′-GUGUAAGGCUGUCCAUAUC-3′, 703si and 5′-GUGU UUCAAUUCCAGCAUA-3′) [25] and scrambled (Scr) siRNA (5′-UUCUCCGAACG UGUCAC GUdTdT-3′ and 5′-ACGUGACACGUUCGGAGAAdTdT-3′) were designed and validated in vitro and in vivo. Intrathecal injection of siRNA, mimics, inhibitor (20 μM in 5 μl of saline), or 3 μl CXCR4 antagonist AMD3100 (0.005 mg/μl; ab141825, Abcam) was performed at spinal L3–L5 levels of the mouse. Overall, daily intrathecal injections of siRNA for 3 consecutive days or mimics, inhibitor, and scrambled control for 2 consecutive days were performed in naïve or pSNL (beginning 7 days after pSNL) mice before sample collection or further behavior tests to allow sufficient overexpression or inhibition. AMD3100 was injected once after pSNL or administration of overexpression/inhibition reagents. Otherwise, please see specified injection time points detailed in corresponding figure legend of Fig. 2.

### miR-23a target construction

The 3′-untranslated region (3′-UTR) sequence of miR-23a targeting CXCR4 was synthesized [wild-type(wt)-Cx, 5′-P-TCGAAATACTTTTTTTTGTTTGTTTGTTTCAT GTGAATGAGTGTCTAGGCAGGACCTGT-3′, and 5′-P-GGCCACAGGTCCTGCCTA GACACTCATTCACATGAAACAAACAAACAAAAAA AAGTATT-3′; mutation type (mut)-Cx, 5′-P-TCGAAATACTTTTTTTTGTTTGTTTGTTTCAGGAAAATGAGTGT CTAGGCAGGACCTGT-3′, and 5′-P-GGCCACAGGTCCTGCCTAGACACTCATTTT CCTGAAACAAACAAACAAAAAAAAGTATT-3′]. The annealed forward and reverse oligos of wt-Cx and mut-Cx were respectively inserted into a dual reporter psiCHECK2 plasmid digested by Xhol and NotI and named as wild-type CHK-wt-CXCR4 and mutant-type CHK-mut-CXCR4.

### Immunofluorescence and fluorescence in situ hybridization

The procedure was performed as described in a previous study [24]. Briefly, segments of spinal L3–L5 were rapidly dissected from mice perfused with 4% paraformaldehyde, fixed with 4% PFA, then cryoprotected in 30% sucrose. For double-labeling immunofluorescence (IF) analysis, the spinal sections were incubated overnight at 4 °C with a mixture of NeuN antibody (1:100, MAB377; Millipore), GFAP antibody (1:500, ab10062, ab7260, Abcam), IBA1 antibody (1:200, WAKO, 019-19741), and CXCR4 antibody (1:100; ab124824, Abcam) or VDUP-1 antibody (TXNIP, 1:50; sc-271238, Santa Cruz Biotechnology). The sections were then incubated with fluorescent-conjugated secondary antibody (A11010, R37116, A21206; A10042, Invitrogen) at 37 °C for 1 h. For fluorescence in situ hybridization (FISH) and IF co-staining, CXCR4 antisense or sense probe (CDS: 356-916 in NM_009911.3) or TXNIP antisense or sense probe (883–1185 in NM_001009935.3) with digoxin modification were hybridized to spinal slices as instructed in the FISH kit (Guangzhou Exon) and incubated with fluorescent-conjugated EGFP secondary anti-digoxin and then incubated with NeuN antibody (1:500, MAB377; Millipore), GFAP antibody (1:500, ab10062, ab7260, Abcam), and IBA1 antibody (1:200, WAKO, 019–19,741), respectively, and finally incubated with AlexaFluor 568 antibody (Abcam). After the sections were rinsed in 0.01 M PBS, the slides were mounted on a mounting medium with DAPI and scanned by Zeiss LSM 780 confocal microscope (Germany). One section every 8 sections (120 μm) in a series of 24 sections was chosen to process the same pairs of antibody immunostaining in L3–L4 segment. All double-labeling immunostaining assessments for each pair of protein targets in the spinal cord were repeated in L3–L4 and L4–L5 segments of one specimen. Six sections with Z stack were taken for analysis.

The quantification of double-labeled cells was analyzed as previously described [26]. Take the percentage calculation of CXCR4-positive cells in neurons as an example, the NeuN(+) cells and NeuN(+)/CXCR4 (+) cells in lamina I–IV of six sections with Z stack of the L3–L5 spinal dorsal horn of each mouse were counted manually, and then, the number of NeuN(+)/CXCR4(+) cells were divided by the number of NeuN(+) cells in each section. The resulting value of six sections was then averaged, representing the data of one specimen. Each group had five animals. All analyses were performed by an investigator who was initially blinded to treatment groups.

### Immunoprecipitation

Immunoprecipitations (IPs) were performed using the Catch and Release Reversible Immunoprecipitation System kit (Millipore) according to the manufacturer’s instructions. Antibodies used for IPs were as the following: TXNIP-1 (sc271238, Santa Cruz Biotechnology) and CXCR4 (ab124824, Abcam). Rabbit/mouse IgG were used as control for co-immunoprecipitation (IP) assays.

### Mammalian two-hybrid assay

For the mammalian two-hybrid assay, plasmids pBIND (encoding the yeast GAL4 DNA binding domain upstream of a multiple cloning region, MCR) and pACT (encoding the herpes simplex virus VP16 activation domain upstream of a MCR and expressing the *Renilla reniformis* luciferase) and the reporter plasmid encoding firefly luciferase (pG5Luc) were purchased from Promega (CheckMate Mammalian Two-Hybrid System). The expression plasmids were constructed according to the scheme shown in Fig. 4d. To generate pBIND-TXNIP, a fusion protein of GAL4 DNA binding domain and coding sequence of TXNIP, the coding sequence of TXNIP was amplified by PCR from cDNA of mouse spinal cord and inserted into the MCR of pBIND vector. The C-terminal of CXCR4 was also amplified by PCR from cDNA of mouse spinal cord and, respectively, fused to the VP16 domains of the expression plasmid pACT at the BamH1-EcoR V site to construct pACT-Cxcr4-C fusion activation expression vector. All constructs were verified by PCR and DNA sequencing.

### Luciferase reporter assay

HEK293T cells were cultured in DMEM with 10% FBS. HEK293T cells were seeded at 1 × 10^5^ cells per 24-well. Identification of miR-23a targets was performed by transfecting CHK-wt-CXCR4 or CHK-mut-CXCR4 plasmids (50 ng) and miR-23a mimics (80 ng) or inhibitor (50 ng) into HEK293T cells using Lipofectamine 2000 (11668-027, Invitrogen) in a 24-well plate. Cell lysates were prepared and subjected to luciferase assays using the Dual-Luciferase reporter kit (E1910, Promega) at 48 h after transfection according to the manufacturer’s instruction. For luciferase activity test in mammalian two-hybrid, following the manufacturer’s instructions, both or only one of the pGAL4 and pVP16 fusion constructs (pBIND-TXNIP and pACT-CXCR4-C) were co-transfected with the pG5luc Vector into 293T cells using Lipofectamine 2000 (11668-027, Invitrogen) with a molar ratio of 1:1:1 for pACT-CXCR4-C:pBIND-TXNIP:pG5luc Vector. After incubation for 48 h, luciferase activity was measured using the Dual-Luciferase reporter kit (E1910, Promega). Results were expressed as firefly luciferase activity to Renilla luciferase activity ratio (*F*/*R*). The *F*/*R* ratio was calculated using the following formula: *F*/*R* mean of firefly luciferase activity/mean of Renilla luciferase activity.

### Western blot analysis

Protein (20–50 μg) was extracted from the ipsilateral spinal cord dorsal horn and separated with 10% SDS-PAGE gel, transferred onto a nitrocellulose membrane, and incubated with antibody against CXCR4 (1:1000; ab124824, Abcam), VDUP-1 (1:1000; sc-271238, Santa Cruz Biotechnology), NLRP3 (1:500; NBP1-77080, Novus Biologicals), apoptosis-associated speck-like molecule containing CARD domain (ASC; 1:1000; sc-22514-R, Santa Cruz Biotechnology), Caspase1 (1:1000; ab-1872, Abcam), Caspase1 p10 (1:1000; sc-514, Santa Cruz Biotechnology), and IL-1β (1:1000; ab9722, Abcam) or control β-Actin (1:5000; YM3028, ImmunoWay). Blots were washed and incubated in HRP-linked anti-rabbit IgG antibody (1:5000; 7074, Cell Signaling Technology) and HRP-linked anti-mouse IgG antibody (1:5000; 7076, Cell Signaling Technology). Protein blots were visualized using Clarity ECL Substrates (Biorad).

### Statistical analysis

Data are presented as mean ± SEM and were analyzed using GraphPad Prism v5.00. The results were statistically analyzed with a one-way or two-way ANOVA or paired or unpaired Student’s *t* test. When ANOVA showed a significant difference, pairwise comparisons between means were tested by the post hoc Tukey method. A *p* value of less than 0.05 was considered statistically significant.

## Results

### Spinal glial CXCR4 contributes to pSNL-induced neuropathic pain

CXCR4 has been identified as an inflammatory-related regulatory factor in mammals. As the spinal cord plays critical roles in transducing and transmitting pain-related gene signaling, we explored the potential modulatory role of spinal CXCR4 in nociceptive response. We first examined the changes in levels of spinal CXCR4 in pSNL-induced nociception. Immunofluorescence staining showed only a 23.5% overlap between the CXCR4 signal and NeuN (a neuron marker) in the sham group and 10.3% overlap in the pSNL group (Fig. 1a, a′); however, CXCR4 had 33.3% in the sham group and 58% overlap in the pSNL group with GFAP (an astrocyte marker), respectively (Fig. 1b, b′), and 66.7% in the control group and 87% in the pSNL group overlap with IBA1 (a microglial marker), respectively (Fig. 1c, c′). GFAP and especial IBA1-positive cells were increased in the spinal cord of pSNL mice (Fig. 1b, c), indicating the activation of both astrocyte and microglia after nerve injury. These results suggested that pSNL injury induced the increase of CXCR4 and mainly occurred in spinal astrocytes and microglial cells. Furthermore, to confirm the CXCR4 expression in spinal cell types, we designed the CXCR4 mRNA antisense probe and negative control sense probe then performed co-staining of CXCR4 mRNA fluorescence in situ hybridization (FISH) and above three protein marker immunofluorescence in normal mouse spinal cord. The co-staining results showed a 27% overlap between the CXCR4 signal and NeuN, 52% between the CXCR4 signal and GFAP, and 57% between CXCR4 and IBA1 by the use of antisense probe and, however, no fluorescent signals by the use of sense probe (data not shown), suggesting the distribution of CXCR4 not only in spinal neuron, but also in spinal astrocyte and microglial cells (Additional file 1: Figure S1). Additionally, the levels of CXCR4 were markedly increased on day 7 post-pSNL compared to those after sham surgery in the ipsilateral dorsal horn of mice (Fig. 1a–c); however, no signal was observed in isotype control with anti-CXCR4 (data not shown). We then determined the role of CXCR4 in neuropathic pain by inhibiting CXCR4 with AMD3100, a specific antagonist of CXCR4 [15]. Consistent with our previous behavior tests [5], inhibition of CXCR4 with AMD3100 reversed the thermal hyperalgesia and mechanical allodynia, respectively, at 1 and 2 h after injection of AMD3100 in pSNL mice (Fig. 1d, e). Considering lentivirus works well in several cells such as neurons [27] and microglial [28] and astrocyte cell [29], we employed the lentivirus to mediate the expression of target gene in the present study. With lentivirus-mediated expression, overexpression of CXCR4 with Lenti-CXCR4 produced the hypersensitivity for thermal and mechanical stimulus, respectively, on day 5 and day 9 after intrathecal injection in naïve mice (Fig. 1f, g). These results suggest that the increased spinal glial CXCR4 has an essential role in the pathogenesis of neuropathic pain.

### Spinal miR-23a regulates CXCR4 expression in pSNL-induced neuropathic pain

We recently reported that miRNAs play a key role in the regulation of pain behavior [27]. However, it is still unknown whether miRNA could regulate neuropathic pain by targeting CXCR4. We firstly predicted the potential miRNAs regulating CXCR4 by the use of two independent programs: 19 miRNAs from TargetScan (http://www.targetscan.org) and 26 miRNAs from MicroRNA (http://www.microrna.org). A total of 13 shared miRNAs were harvested with species conservation binding sites to CXCR4 among vertebrates, such as rats, chimps, and humans (Fig. 2a). Among them, miR-23a predictably regulates CXCR4 by binding to 202–208 region of 3′UTR in CXCR4 mRNA (Fig. 2b). Then, we evaluated the temporal expression pattern of miR-23a in the spinal cord. qRT-PCR results showed that no significant alteration of miR-23a expression was found in the sham group. However, compared with the sham group, miR-23a expression was significantly decreased from 1 to 7 days after pSNL surgery in pSNL group, but it slightly recovered on day 21 (Fig. 2c). These results suggest that the downregulation of spinal miR-23a after pSNL injury, which subsequently reduces its inhibitory effect on CXCR4, may be required for the maintenance of neuropathic pain.

To experimentally validate the in silico prediction, we constructed luciferase reporter vectors containing CXCR4-3′UTR region recognized by miR-23a. Co-transfection of miR-23a mimics with the wild-type reporter CHK-wt-CXCR4 decreased luciferase activity by 41% compared with the mutation-type reporter CHK-mut-CXCR4. In contrast, miR-23a inhibitor increased the luciferase activity by 30% in CHK-wt-CXCR4 but not in CHK-mut-CXCR4 (Fig. 2d). Furthermore, we investigated the regulatory role of miR-23a in CXCR4 expression in vivo. Intrathecal injection of miRNA mimics (miR-23a mimics) or lentivirus (Lenti-miR-23a) upregulated the expression of spinal miR-23a. The transfection efficiency of Lenti-miR-23a in the mouse spinal cord was validated by qRT-PCR. Spinal miR-23a was increased respectively by 91 or 95% after intrathecal injection of miR-23a mimics or Lenti-miR-23a for 2 continuous days in naïve mice, and decreased miR-23a was reversed by the intrathecal injection of miR-23a mimics or Lenti-miR-23a for 2 continuous days in pSNL mice with 7-day surgery (Fig. 2e). Furthermore, we found that the pSNL-induced CXCR4 protein was decreased by 20.1% after miR-23a mimic injection (Fig. 2f) and by 22.8% after Lenti-miR-23a injection (Fig. 2g), respectively, but not by scrambled miRNA or lentivirus vector control. Additionally, spinal CXCR4 expression was increased by 37.6 or 39.4%, respectively, after miR-23a knockdown with miR-23a inhibitor (Fig. 2h) or LV-miR-23a (expressing two molecules of miR-23a antisense, a “miRNA-loss-of-function” strategy) (Fig. 2i) in naïve mice. The in vitro and in vivo findings suggest that miR-23a, through directly binding to CXCR4-3′UTR, regulates the expression of spinal CXCR4 following pSNL-induced neuropathic pain.

### Spinal miR-23a regulates neuropathic pain via CXCR4

**Fig. 3:**
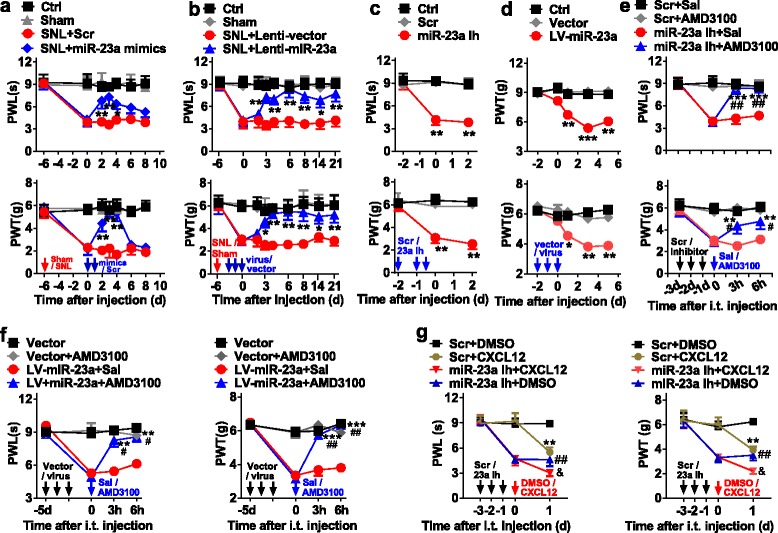
Spinal miR-23a regulates pain behavior via CXCR4. **a**, **b** Daily intrathecal injections of miR-23a mimics (**a**) or Lenti-miR-23a (**b**) for 2 or 3 consecutive days, respectively, reversed pSNL-induced thermal hyperalgesia and mechanical allodynia. Two-way ANOVA (effect versus group × time interaction) followed by post hoc Tukey test, **a** PWL *F*_group_ (18, 112) = 10.24, PWT *F*_group_ (18, 112) = 12.02; **b** PWL *F*_group_ (18, 112) = 10.57, PWT *F*_group_ (18, 112) = 8.12, *p < 0.05, **p < 0.01 versus pSNL+Scr or pSNL+Lenti-vector group. **c**, **d** Daily intrathecal injections of **c** miR-23a inhibitor or **d** LV-miR-23a for 2.5 or 3 consecutive days, respectively, produced thermal hyperalgesia and mechanical allodynia in naïve mice. Two-way ANOVA (effect versus group × time interaction) followed by post hoc Tukey test, **c** PWL *F*_group_ (4, 36) = 26.64, PWT *F*_group_ (4, 36) = 24.42; **d** PWL *F*_group_ (8, 60) = 4.83, PWT *F*_group_ (8, 60) = 5.31, **p < 0.01, ***p < 0.001 versus vector group. **e**, **f** Blocking CXCR4 with AMD3100 significantly reversed thermal hyperalgesia and mechanical allodynia induced by miR-23a inhibitor (**e**) or LV-miR-23a (**f**) in naïve mice. Two-way ANOVA (effect versus group × time interaction) followed by post hoc Tukey test, **e** PWL *F*_group_ (9, 64) = 27.84, PWT *F*_group_ (9, 64) = 12.69; **f** PWL *F*_group_ (9, 64) = 11.10, PWT *F*_group_ (9, 64) = 14.55, **p < 0.01, ***p < 0.001 versus 0 h before AMD3100 injection. ^#^*p* < 0.05, ^##^*p* < 0.01 versus miR-23aIh + Sal or Lv-23a + Sal. Red arrow indicates pSNL surgery day. Blue or black arrow indicates injection time points of specified drug. Data are presented as mean ± SEM; *n* = 5 per group. **g** Intrathecal injection of CXCL12 further increased the thermal and mechanical sensitivity induced by miR-23a knockdown, with miR-23a inhibitor, in naïve mice. PWL *F*_group_ (6, 48) = 31.74, PWT *F*_group_ (6, 48) = 21.87; **p < 0.01 versus Scr + DMSO. ^##^*p* < 0.01 versus Scr + DMSO. ^&^*p* < 0.05 versus miR-23a Ih + CXCL12. Black arrow indicates miR-23 inhibitor or Src injection. Red arrow indicates injection time points of CXCL12 or control DMSO. Data are presented as mean ± SEM; *n* = 5 per group

To investigate whether miR-23a contributes to the modulation of neuropathic pain via CXCR4, we tested the changes of pain behavior after manipulating spinal miR-23a. The results showed that intrathecal injection of miR-23a mimics or Lenti-miR-23a, but not control scrambled miRNAs or Lenti-vector, for 2 or 3 consecutive days, significantly reversed pSNL-induced thermal hyperalgesia and mechanical allodynia (Fig. 3a, b). In contrast, in naïve mice, downregulation of miR-23a via intrathecal injection of miR-23a inhibitor or LV-miR-23a, but not scrambled miRNAs or vector, for 3 consecutive days, produced pain-like behavior (Fig. 3c, d). These findings suggest that spinal miR-23a is involved in the process of neuropathic pain.

To determine the role of CXCR4 in miR-23a-mediated modulation of neuropathic pain at the behavioral level, we inhibited CXCR4 with AMD3100 in the presence of miR-23a inhibition with miR-23a inhibitor (Fig. 3e) or LV-miR-23a (Fig. 3f), followed by behavioral tests. The results showed that antagonizing CXCR4 alleviated miR-23a inhibition-induced thermal hyperalgesia and mechanical allodynia in naïve mice (Fig. 3f), indicating that CXCR4 acts as the downstream effector of miR-23a-mediated modulation of pain behavior. However, motor function was not affected by the manipulation with miR-23a (data not shown). CXCL12 could activate CXCR4 via binding and contribute to the generation of pain behavior [5]. Therefore, we determined whether intrathecal injection of CXCL12 strengthened the pain-like behavior induced by miR-23a knockdown. The behavior testing showed that single CXCL12 increased the sensitivity to thermal or mechanical stimulus on day 1 after intrathecal injection in naïve mice, compared to the DMSO control group (Fig. 3g). And combining to treat the mice with CXCL12 further aggravated the pain sensitivity induced by knockdown of miR-23a with inhibitor, evidenced by the enhanced thermal hyperalgesia and mechanical allodynia (Fig. 3g), suggesting the down-modulation of miR-23a inducing overexpression of CXCR4 is functional and contributes to pain production. Together, these results suggest that spinal miR-23a regulates neuropathic pain by targeting CXCR4 expression at the spinal level.

### Direct interaction of TXNIP and CXCR4 is regulated by miR-23a in neuropathic pain process

**Fig. 4:**
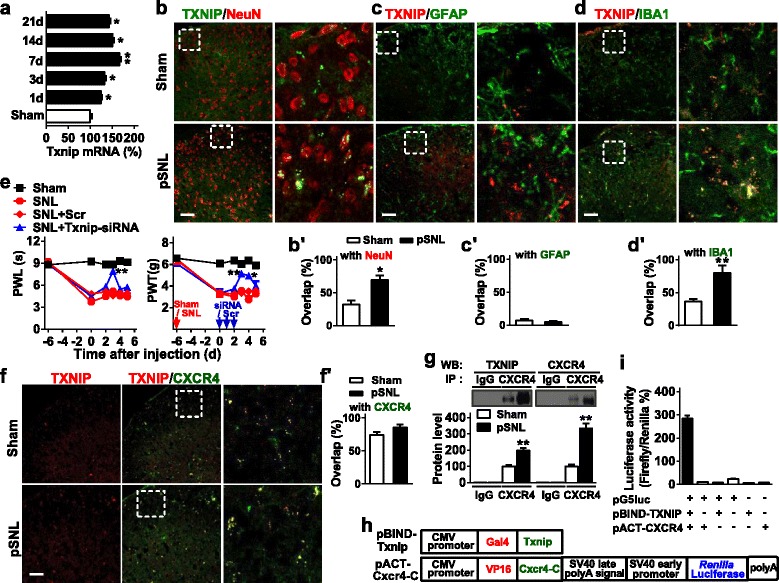
TXNIP is involved in the process of neuropathic pain by the CXCR4-dependent regulation. **a** Quantitative expression of Txnip mRNA at 1, 3, 7, 14, and 21 days after pSNL surgery. One-way ANOVA (expression versus time point) followed by post hoc Tukey test, *F*_time_ (5, 24) = 40.4, *p < 0.05, **p < 0.01 versus sham group; *n* = 5 per group. **b**–**d** TXNIP immunofluorescent co-staining with NeuN (**b** and **b′**), GFAP (**c** and **c′**), or IBA1 (**d** and **d′**) in the lumbar of spinal cord at 7 days after pSNL surgery. *p < 0.05, **p < 0.01 versus sham group by two-tailed unpaired Student’s *t* test; *n* = 5 per group. Scale bar, 50 μm. **e** Daily intrathecal injections of TXNIP siRNA for 3 consecutive days reversed pSNL-induced thermal hyperalgesia and mechanical allodynia. Two-way ANOVA (effect versus group × time interaction) followed by post hoc Tukey test, PWL *F*_group_ (10, 72) = 20.76; PWT *F*_group_ (10, 72) = 6.85, *p < 0.05, **p < 0.001, versus pSNL+Scr group; *n* = 5 per group. **f**, **f′** Co-staining of double immunofluorescence (CXCR4, green; TXNIP, red) in the lumbar segment of the spinal cord of naïve mice. Scale bar, 50 μm. **g** The representative immunoblot demonstrates the interaction of TXNIP and CXCR4 in a co-IP experiment. Rabbit IgG is used as control for co-IP assays. **p < 0.01, ***p < 0.001 versus the corresponding sham group by two-tailed unpaired Student’s *t* test; *n* = 5 per group. **h** The CDS region of gene Txnip was inserted into pBIND to produce Gal4-Txnip chimeric fusion expression vector (pBIND-Txnip), and the C-terminal of Cxcr4 was inserted into pACT to produce VP16-Cxcr4-C chimeric fusion expression vector (pACT-CXCR-C). **i** pBIND-Txnip and/or pACT-Cxcr4-C transfected into 293T cells with the reporter gene vector pG5luc or empty vector. At 48 h after transfection, the interactions were measured by relative luciferase activity. In general, only pBIND-Txnip and pACT-Cxcr4-C co-transfection with pG5luc indicated strong luciferase activity. One-way ANOVA (expression versus the treated groups) followed by post hoc Tukey test, *F* (5, 24) = 628, ***p < 0.001; *n* = 3 per group

TXNIP is an endogenous negative regulator of thioredoxin, a major ubiquitously expressed thiol-reducing non-enzymatic antioxidant. To investigate whether TXNIP was implicated in the pain process, we first examined the expression of TXNIP in the spinal cord. As shown in Fig. 4, TXNIP mRNA was increased from day 3 to day 7 after pSNL injury and peaked on day 7 and kept a relative stable expression after day 7 till day 21 (Fig. 4a). Consisting with the above staining of spinal glial cell, nerve injury increased the astrocyte and microglial positive cells and double staining indicated that TXNIP was co-expressed with NeuN and increased from 32.1% overlap in sham group to 69.5% overlap in pSNL group (Fig. 4b. b′) or that with IBA1 and increased from 36.5% overlap in sham group to 80.5% overlap in pSNL group (Fig. 4d, d′) but almost no overlap with GFAP (Fig. 4c, c′) in the spinal cord, suggesting that TXNIP is majorly expressed in the spinal neurons and spinal microglial cells (Fig. 4d, d′). The further FISH-immunofluorescent co-staining by using TXNIP mRNA antisense probe showed a 33.3% overlap between TXNIP mRNA and NeuN, 66.7% overlap between TXNIP mRNA and IBA1, but hardly any overlap between TXNIP mRNA and GFAP (Additional file 1: Figure S2); however, the fluorescent signal was not observed in the sense probe hybridization (data not shown), confirming immunostaining results about localization and quantification of TXNIP in the spinal dorsal horn. Furthermore, we designed the two siRNAs (681-704 and 1271-1291 in mRNA) and intrathecally injected them into mice, then determined the mRNA and protein level of TXNIP. We found that treatment with siRNA-681, but not siRNA-1271, significantly knocked down the expression of TXNIP in mRNA and protein (Additional file 1: Figure S3). Correspondingly, knockdown of TXNIP with intrathecal injection of 681-siRNA for 3 consecutive days significantly alleviated the thermal hyperalgesia and mechanical allodynia induced by pSNL (Fig. 4e), suggesting that TXNIP contributes to the processing of pSNL-induced neuropathic pain.

As CXCR4 was expressed not only in the spinal astrocytes but also in spinal microglia, we further want to know whether CXCR4 regulates neuropathic pain by its potential interaction with TXNIP. First, we investigated whether TXNIP and CXCR4 were co-expressed in the spinal cord. Results from double staining revealed that TXNIP signal had 74.3% overlap with CXCR4 in spinal lumbar dorsal horn of sham group and 85.1% overlap with CXCR4 in that of pSNL group (Fig. 4f, f′). Then, we examined whether CXCR4 bound to the TXNIP protein, as expected, co-IP identified the formation of CXCR4 and TXNIP complex in the spinal cord, and the number of this complex was increased after pSNL; however, CXCR4 or TXNIP was not detected in samples from IgG group, indicating the IP specificity of CXCR4 antibody or TXNIP antibody (Fig. 4g). Furthermore, we carried out an in vitro study using the mammalian two-hybrid assay to identify whether there was a direct functional interaction between CXCR4 and TXNIP. In the vector construction, the CDS region of TXNIP gene was inserted into pBIND to generate Gal4-TXNIP chimeric fusion expression plasmid (pBIND-TXNIP), and C-terminal of CXCR4 was cloned into pACT to generate VP16-CXCR4-C chimeric fusion expression plasmid (pACT-CXCR4-C; Fig. 4h). The interaction between CXCR4 and TXNIP was determined by co-transfection of the reporter gene vector pG5luc with pBIND-TXNIP and pACT-CXCR4-C. After co-expression of pBIND-TXNIP and pACT-CXCR4-C in 293T cells, their interaction was measured through the luciferase activity of reporter gene. The pBIND and pACT were used as negative control plasmids. The results showed that transcription of the reporter vector was significantly activated through the co-expression of pBIND-TXNIP and pACT-CXCR4-C. No significant activation was observed in the co-transfection of reporter vector pG5luc and one of the chimeric fusion expression vectors, pBIND-TXNIP or pACT-CXCR4-C (Fig. 4i). These results indicate a direct interaction between CXCR4 and TXNIP in neuropathic pain.

**Fig. 5:**
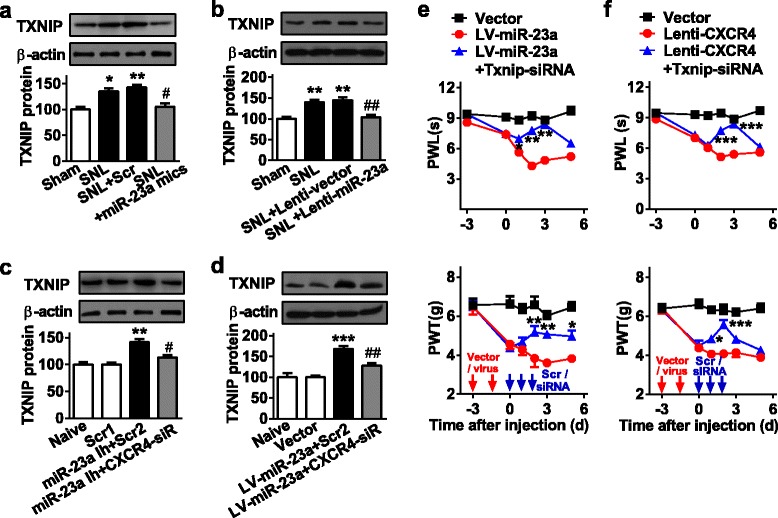
miR-23a/CXCR4 regulates neuropathic pain via TXNIP. **a**, **b** Increased spinal TXNIP protein expression in pSNL mice was reversed by intrathecal injection of miR-23a mimics (**a**) or Lenti-miR-23a (**b**). Intrathecal injections began on day 7 after pSNL. TXNIP was measured at 24 h after 2-day miR-23a mimics injections or at 48 h after 3-day Lenti-miR-23a injections. One-way ANOVA (expression versus treatment groups) followed by post hoc Tukey test **a** *F* (3, 16) = 12.54; **b** *F* (3, 16) = 17.27, *p < 0.01, **p < 0.01 versus sham group. ^#^*p* < 0.05, ^##^*p* < 0.01 versus pSNL+Scr or pSNL+Lenti-vector group. *n* = 5 per group. **c**, **d** TXNIP protein expression increased by intrathecal injections of miR-23a Ih (**c**) or LV-miR-23a (**d**) in naïve mice was inhibited by intrathecal injection of CXCR4 pool siRNA. Intrathecal injection of CXCR4 pool siRNA was performed at 24 h after miR-23a Ih injections for 2 consecutive days or 48 h after LV-miR-23a injections for 3 consecutive days. TXNIP was measured at 24 h after CXCR4 pool siRNA injection. One-way ANOVA (expression versus treatment groups) followed by post hoc Tukey test **c** *F* (3, 16) = 18.1; **d** *F* (3, 16) = 31.69, **p < 0.01, ***p < 0.001 versus vector group. ^##^*p* < 0.01 versus miR-23a Ih or LV-miR-23a group. *n* = 5 per group. **e** Daily intrathecal injections of TXNIP siRNA for 3 consecutive days reversed pain-like behavior induced by LV-miR-23a. Two-way ANOVA (effect versus group × time interaction) followed by post hoc Tukey test, PWL *F*_group_ (10, 72) = 13.05; PWT *F*_group_ (10, 72) = 3.68. *p < 0.05, **p < 0.001 versus Lenti-miR-23a group; *n* = 5 per group. **f** Daily intrathecal injections of TXNIP siRNA for 3 consecutive days reversed pain-like behavior induced by Lenti-CXCR4. Two-way ANOVA (effect versus group × time interaction) followed by post hoc Tukey test, PWL *F*_group_ (10, 72) = 20.76; PWT *F*_group_ (10, 72) = 6.85, *p < 0.05, ***p < 0.0001 versus Lenti-CXCR4 group; *n* = 5 per group. Red arrow indicates pSNL day or vector/virus injection time points. Blue arrow indicates injection time points of Scr/siRNA. Data are presented as mean ± SEM

As we have shown that miR-23a regulates CXCR4 and that CXCR4 interacts with TXNIP in neuropathic pain, it is possible that miR-23a may regulate TXNIP via CXCR4 in neuropathic pain. We found that the pSNL-induced spinal TXNIP expression was decreased by 35.8 and 39.2% with miR-23a overexpression by intrathecal injection of miR-23a mimic and Lenti-miR-23a, respectively, but not affected by scrambled control or lentivirus vector (Fig. 5a, b). In contrast, TXNIP expression was significantly enhanced in the spinal cord of naïve mice with miR-23a inhibition by intrathecal injection of miR-23a inhibitor or LV-miR-23a, whereas no changes were observed by intrathecal injection of scrambled or empty vector (Fig. 5c, d). Furthermore, we determined whether CXCR4 knockdown could inhibit the increase of TXNIP induced by miR-23a overexpression. Firstly, the efficiency of CXCR4 pool siRNAs [25] was validated in naïve mice or pSNL mice. We found that CXCR4 was markedly decreased in naïve mice or the increased CXCR4 was reversed in pSNL-induced neuropathic pain mice 2 days after intrathecal injection of CXCR4-siRNA (data not shown). Then, we intrathecally post-injected mice with the increase of TXNIP induced by knockdown of miR-23a; the result showed that miR-23a inhibition-induced increase of TXNIP expression was markedly reduced by CXCR4 knockdown with siRNA, suggesting that miR-23a regulates TXNIP expression via CXCR4 at the protein level (Fig. 5c, d). To further determine the role of TXNIP in miR-23a/CXCR4-mediated pain process at the behavioral level, we post-treated the animals with siRNA to knockdown TXNIP in the presence of miR-23a inhibition or CXCR4 overexpression with respective intrathecal injection of LV-miR-23a or Lenti-CXCR4 and then observed the behavioral response. Knocking down of TXNIP markedly alleviated the hypersensitivity to thermal and mechanical stimulus induced by miR-23a inhibition (Fig. 5e) or CXCR4 overexpression (Fig. 5f) in naïve mice. These data suggest that TXNIP acts as one of the downstream effectors of miR-23a/CXCR4-mediated modulation of neuropathic pain.

### miR-23a/CXCR4 regulates neuropathic pain by modulation of NLRP3 inflammasome via TXNIP

**Fig. 6:**
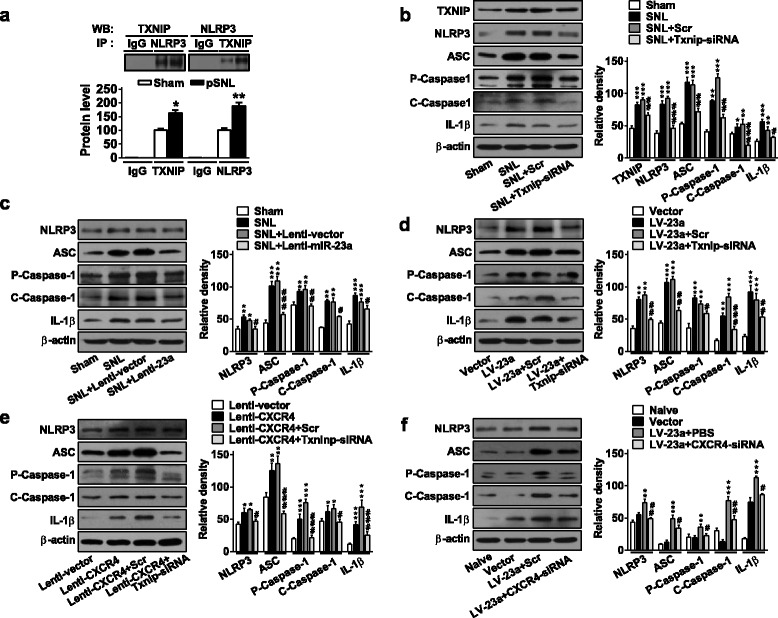
TXNIP regulates the protein expression of spinal NLRP3 inflammasome in SNL-induced neuropathic pain. **a** Co-IP of TXNIP and NLRP3. Rabbit IgG is used as control for co-IP assays. *p < 0.05, **p < 0.01 versus the corresponding sham group by two-tailed unpaired Student’s *t* test; *n* = 5 per group. **b** Increased NLRP3 inflammasome complex after pSNL, including NLRP3, ASC, P-Caspase1, C-Caspase1, and mature IL-1β, were reversed by knockdown of TXNIP with siRNA. Inflammasome complex expression was determined on day 7 after pSNL or at 24 h after 3-day injections of TXNIP siRNA or Scr, beginning on day 7 after pSNL. One-way ANOVA (expression versus the treated groups) followed by post hoc Tukey test, *F* (3, 8) = 76.56 for TXNIP, 106.9 for NLRP3, 79.01 for ASC, 191.3 for P-Caspase1, 26.68 for C-Caspase1, and 33.34 for mature IL-1β, **p < 0.01, ***p < 0.001 versus sham group. ^#^*p* < 0.05, ^##^*p* < 0.01, and ^###^*p* < 0.001 versus pSNL+Scr group. **c** Increased NLRP3 inflammasome were reversed by intrathecal injection of Lenti-miR-23a in pSNL mice. Inflammasome complex expressions were measured at 7 days after pSNL surgery or at 48 h after 3-day injections of Lenti-miR-23a or Lenti-vector, beginning on day 7 after pSNL. One-way ANOVA (expression versus the treated groups) followed by post hoc Tukey test, *F* (3, 8) = 20.79 for NLRP3, 96.78 for ASC, 29.35 for P-Caspase1, 106.1 for C-Caspase1, and 42.21 for mature IL-1β, **p < 0.01, ***p < 0.001 versus sham group. ^#^*p* < 0.05, ^##^*p* < 0.01, and ^###^*p* < 0.001 versus pSNL+Lenti-miR-23a group. **d** LV-miR-23a-induced expression of NLRP3 inflammasome was reversed by knockdown of TXNIP with siRNA in naïve mice. Content of inflammasome was examined at 48 h after 3-day injections of LV-miR-23a or at 24 h after 3-day injections of TXNIP siRNA or Scr (starting after the injections LV-miR-23a or vector). One-way ANOVA (expression versus the treated groups) followed by post hoc Tukey test, *F* (3, 8) = 106.7 for NLRP3, 138.4 for ASC, 54.04 for P-Caspase1, 133 for C-Caspase1, and 50.59 for mature IL-1β, **p < 0.01, ***p < 0.001 versus vector group. ^#^*p* < 0.05, ^##^*p* < 0.01, and ^###^*p* < 0.001 versus LV-miR-23a + Scr group. **e** Increased NLRP3 inflammasome induced by injection of Lenti-CXCR4 was reversed by injection of TXNIP siRNA in naïve mice. Inflammasome complex expressions were measured at 48 h after 3-day injections of Lenti-CXCR4 or at 24 h after 3-day injection of TXNIP siRNA or Scr (starting after injections of Lenti-CXCR4 or Lenti-vector). One-way ANOVA (expression versus the treated groups) followed by post hoc Tukey test, *F* (3, 8) = 18.84 for NLRP3, 48.48 for ASC, 84.25 for P-Caspase1, 8.63 for C-Caspase1, and 48.37 for mature IL-1β, **p < 0.01, ***p < 0.001 versus Lenti-vector group. ^#^*p* < 0.05, ^##^*p* < 0.01, and ^###^*p* < 0.001 versus Lenti-CXCR4 + Scr group. **f** NLRP3 inflammasome upregulated by injection of LV-miR-23a was reversed by injection of CXCR4 siRNA in naïve mice. One-way ANOVA (expression versus the treated groups) followed by post hoc Tukey test, *F* (3, 8) = 42.41 for NLRP3, 72.1 for ASC, 10.9 for P-Caspase1, 82.57 for C-Caspase1, and 290.7 for mature IL-1β, **p < 0.01, ***p < 0.001 versus vector group. ^#^*p* < 0.05, ^##^*p* < 0.01 versus LV-miR-23a + Scr group. Expression of inflammasome was measured at 48 h after 3-day injections of LV-miR-23a or at 6 h after injection of CXCR4 siRNA or Scr (beginning after injections of LV-miR-23a or Vector). Data are presented as mean ± SEM; *n* = 3 per group

Recent studies have connected microglial NLRP3 inflammasome with chronic pain behavior [30, 31]. TXNIP is essential for activation of the NLRP3 inflammasome. TXNIP is allowed to bind NLRP3; thus, the activated TXNIP by such reactive oxygen species induced the increase of NLRP3 inflammasome activity. In contrary, TXNIP deficiency impairs the activation of NLRP3 inflammasome and subsequent secretion of interleukin 1beta (IL-1β) [13]. TXNIP/NLRP3 inflammasome axis was identified in the pathologic process of metabolic or CNS injury [32]. Therefore, we further determined whether TXNIP could activate NLRP3 in a protein binding-dependent manner under the neuropathic pain conditions. The co-IP results exhibited that TXNIP did pull down the NLRP3 protein, while NLRP3 pulled down the TXNIP protein, and the formed complex of TXNIP and NLRP3 was increased in the spinal cord of pSNL-induced pain mice; however, TXNIP or NLRP3 was undetected in samples from the IgG group, indicating TXNIP activated the NLRP3 in a protein binding-dependent manner during the neuropathic pain processing (Fig. 6a). To further evaluate whether TXNIP/NLRP3 inflammasome axis was linked to the process of neuropathic pain, we measured the expression of key components of NLRP3 inflammasome including NLRP3, ASC, P-Caspase-1, C-Caspase-1, and mature IL-1β in the spinal cord after manipulations of miR-23a, CXCR4, and TXNIP. Our results showed that pSNL injury increased the protein expression of spinal NLRP3, ASC, P-Caspase-1, C-Caspase-1, and IL-1β compared to that of the sham group, suggesting that spinal NLRP3 inflammasome is associated with the development of neuropathic pain. However, increase of protein expression of the five proteins in NLRP3 inflammasome were reversed by knockdown of TXNIP with intrathecal injection of siRNA in pSNL mice (Fig. 6b), indicating that TXNIP is involved in the regulation of NLRP3 inflammasome in neuropathic pain.

**Fig. 7:**
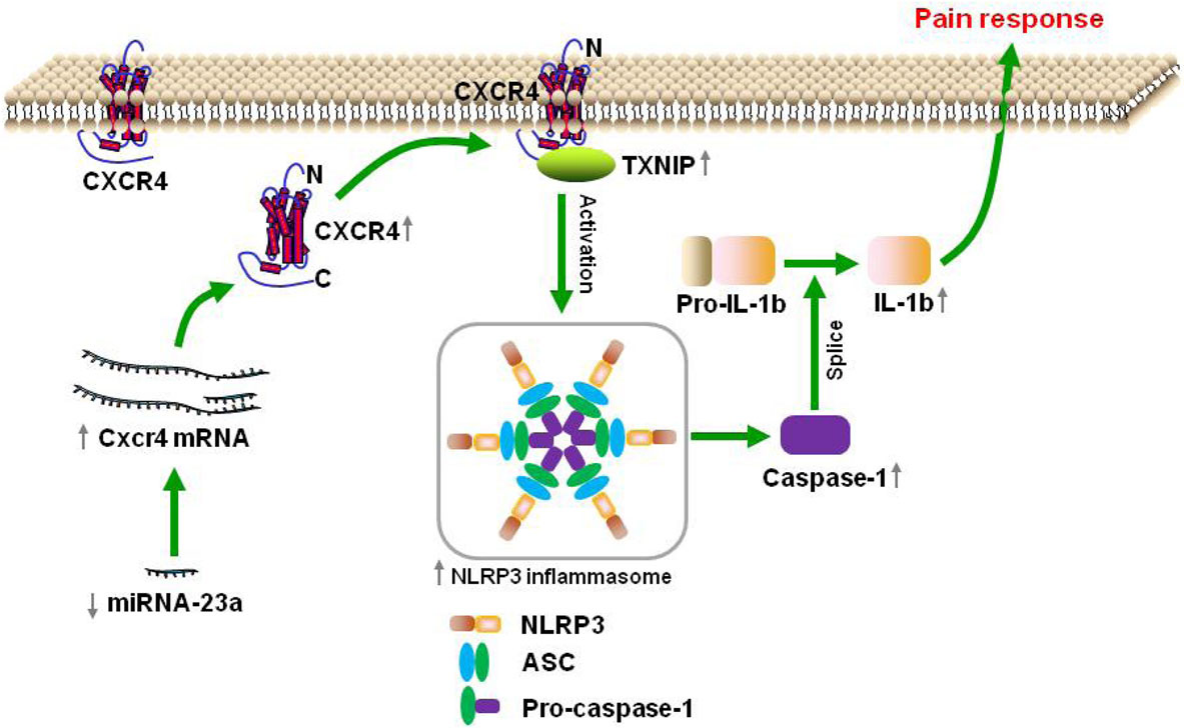
The schematic of miR-23a targeting CXCR4 regulates neuropathic pain via TXNIP/NLRP3 inflammasome in spinal glial cells of mice. In pSNL-induced chronic neuropathic pain, spinal miR-23a expression was significantly reduced, which increased the expression of spinal CXCR4, and subsequently the expression of TXNIP and NLRP3 inflammasome including NLRP3, ASC, Caspase-1, and IL-1β

Based on the fact that miR-23a/CXCR4 regulates neuropathic pain by directly targeting TXNIP and TXNIP regulates the expression of NLRP3 inflammasome in neuropathic pain, we supposed to investigate whether miR-23a or CXCR4 could regulate NLRP3 inflammasome via TXNIP. As shown in Fig. 6c, overexpression of miR-23a significantly reduced the increase of NLRP3 inflammasome evidenced by downregulation of NLRP3, ASC, P-Caspase-1, C-Caspase-1, and IL-1β after pSNL. Contrarily, knockdown of miR-23a with LV-miR-23a or overexpression of CXCR4 by Lenti-CXCR4 increased the expression of spinal NLRP3, ASC, P-Caspase-1, C-Caspase-1, and IL-1β, which were reversed by knockdown of TXNIP with siRNA in naïve mice (Fig. 6d, e). Further, miR-23a knockdown increased expression of spinal NLRP3, ASC, P-Caspase-1, C-Caspase-1, and IL-1β, which were reduced by CXCR4 knockdown with intrathecal injection of siRNA (Fig. 6f). Collectively, these results suggest that miR-23a/CXCR4 modulates neuropathic pain via the TXNIP/NLRP3 inflammasome axis (Fig. 7).

## Discussion

The present study is for the first time to identify a novel role of miR-23a as a regulator of neuropathic pain by directly targeting CXCR4 at spinal level. We further found that TXNIP/NLRP3 inflammasome axis is a direct downstream effector of miR-23a/CXCR4 pathway in spinal glial cells. Our results demonstrate the functional regulation of neuropathic pain by miR-23a via CXCR4/TXNIP/NLRP3 inflammasome.

Neuropathic pain is one of the most intractable human complaints and is caused by lesion or dysfunction of the nervous system [33, 34]. The dysregulation of pain-related gene expression in spinal neuronal or glial cells (astrocytes and microglial cells) is the most prominent contributor in various nociceptive pathways (e.g., in the DRG, spinal cord, and pain-related brain regions) underlying neuropathic pain [5, 35–37]. MiRNAs are important post-transcriptional regulators of gene expression via silencing or degrading mRNA in normal cellular function as well as in pathological processes. Accumulating evidence has shown a strong connection between miRNA (e.g., miRNA-103 [38], let-7b [39], miRNA-7a [40], miRNA-203 [41], miRNA-219 [24], miRNA-365-3p [27], and miRNA-183 cluster [42]) modulation and pain pathways from primary afferent nociceptors, the DRG, the spinal cord, and brain areas in different pain models. Manipulation of miRNA expression prevents and reverses persistent neuropathic, inflammatory, and cancer pain behavior [24, 36, 38]. Interestingly, recent findings suggest that chronic pain is regulated directly by spinal glial miRNA-124 [43], miRNA-29, and miRNA-137 [44], through a combined operation of spinal neuron miRNA-186-5p and glial pain-related gene [36], or extracellularly released miRNA [39]. Additionally, as thermal hyperalgesia and mechanical allodynia are usually used in the measurement of pain behavior, we only investigated the effect of miR-23a on the sensitivity for thermal and mechanical stimulus. Consequently, we found that miR-23a was a potential small RNA in the prevention and inhibition of thermal hyperalgesia and mechanical allodynia induced by injury.

CXCR4 is expressed nearly in all cell types in the peripheral or central nervous system, including neurons, astrocytes, and microglia [1]. CXCR4 not only modulates the neuromodulation, neuroprotection, and neuronal-glial interaction in normal conditions, it is also involved in the neurological disorder caused by human immunodeficiency virus infection, tumor, stroke, and multiple sclerosis [1]. Animals with the deficiency of CXCR4 could not survive due to abnormal tissue development [45]. In 2007, CXCR4 was first found to be involved in the regulation of neuropathic pain induced by the treatment of lysophosphatidylcholine to the gastrocnemius muscle [46]. Furthermore, it was shown that chronic constriction injury (CCI)-induced chronic pain is associated with the increased expression of CXCR4 in neuron and glial cells in bilateral lumbar and cervical DRG [3]. Recently, we firstly reported the essential roles of glial CXCL12/CXCR4 axis in the processing of partial sciatic nerve ligation-induced neuropathic pain [5]. To date, an increasing number of pain studies demonstrate that CXCR4 is associated with nerve injury-induced neuropathic pain [4], cancer pain [47], ischemia-reperfusion-induced pain, diabetic neuropathic pain [48], and opioid tolerance [49] at the DRG or spinal tissues. These findings suggested that the manipulation of CXCR4 is a potential strategy for prevention and therapy of chronic pain. It is noteworthy that both spinal neuronal and glial mechanisms are involved in CXCR4-mediated pain processing, and different species, such as rats and mice, display differential mechanism in spinal cellular types. For example, CXCR4 is co-expressed with NeuN in the spinal cord of cancer-induced bone pain rats [50] or incision-induced postsurgical pain rats [51]. However, CXCR4 is rarely observed in spinal microglial and astrocyte cells of sham rats [51, 52] and significantly increased in spinal microglial and astrocyte cells of ischemia-reperfusion-induced pain rats, further blocking CXCR4 changes in glial membrane receptor, such as TLR4, and inflammatory cytokine release [52], indicating the activation of rat spinal glial cells are associated with the CXCR4. Recent studies demonstrated that CXCR4 is expressed in spinal glia of mice and deletion of mouse glia CXCR4 influences their morphology, mitosis, and progression [53] and regulates spinal cord development [54]. Our findings showed CXCR4 was expressed, not only in spinal astrocyte cells, but also in spinal microglia cells of sham mice, and astrocyte and microglial cells were activated after nerve injury; furthermore, the activation of microglial CXCR4 by astrocyte CXCL12 contributes to the development of neuropathic pain [5]. Interestingly, it seems there is a different distribution of CXCR4 in the spinal cord between CXCR4^+^-GFP transgene mice and normal mice. In CXCR4^+^-GFP transgene mice, CXCR4 is found only in ependymal cells surrounding the central canal of the spinal cord and transfers from the spinal central canal to the spinal periphery after SCI injury; additionally, CXCR4 is found to be co-expressed with microglial but not astrocyte cells [55]. While in non-transgenic mice, CXCR4 is largely expressed throughout the gray and white matter [5]. The difference may be the result of the effect of a transgene tool, such as a vector, on CXCR4 expression. This needs to be further investigated in the future. Indeed, there are reports demonstrating the expression of CXCR4 and its ligand SDF1/CXCL12 in microglial cells and neurons of the DRG and the dorsal horn of the spinal cord [48, 56, 57]. Despite a large body of molecular and functional evidence suggesting that CXCR4 is regulated via extracellular chemokine or its antagonist, it still remains unknown whether CXCR4 is modulated by miRNAs in pain process, and the determination of the subcellular distribution of CXCR4 during the modulation of pain mechanism is needed. In this study, we found that CXCR4 was mainly expressed in spinal microglia and astrocytes. Functionally, miR-23a has a predictive binding capacity to the 3′UTR of CXCR4 mRNA and was decreased significantly in the spinal cord of mice with pSNL-induced neuropathic pain. Interestingly, miR-23a is demonstrated to mediate the post-transcriptional regulation of CXCL12 in human bone marrow stromal cells [58]. Therefore, we wanted to know whether miR-23a regulated CXCR4 expression at the post-transcriptional level in the neuropathic pain. miR-23a was chosen as an experimental target. In fact, miR-23a has been demonstrated to be involved in the pathological process of various diseases, such as adipose metabolism [59], diabetes [60], cancer formation [6], Harada Miuji syndrome [8], inflammation [7], cognitive impairment [9], Alzheimer’s disease [61], and stroke [62]. However, to the best of our knowledge, a functional regulatory role of miR-23a in pain-related CNS disorders has not been reported. Here, we provided the evidence that pSNL-induced neuropathic pain altered miR-23a expression in the spinal cord. Overexpression of spinal miR-23a not just reversed the increase of CXCR4 induced by pSNL, but also alleviated the pain hypersensitivity to thermal and mechanical stimuli. Furthermore, knockdown of miR-23a induced pain-like behavior, accompanied by an increase in CXCR4 expression. As a result, this study elucidated a novel mechanism of miR-23a in the induction and maintenance of neuropathic pain via regulating CXCR4, which expands our knowledge on the functional role of miR-23a in the aforementioned CNS diseases. Notably, considering the contribution of activation of CXCR4 by CXCL12 to nociceptive pain process [5], and CXCL2 as a regulating target of miRNA-23a [58], we intrathecally injected CXCL2 into the mice having pain-like behavior induced by the knockdown of LV-miR-23a. The results exhibited that pain sensitivity behavior is further exaggerated by CXCL12, suggesting either miR-23a alone or synergistic with CXCL12 participated in the regulation and generation of pain behavior following peripheral nerve injury. Additionally, CXCR4 antagonist AMD3100 can elicit analgesic effects and restore the inhibitory neurotransmission, such as GlyRα3 [63], JNK1, and p38 pathways [64], against neuropathic pain. Therefore, we speculated that the decease of miR-23a may affect GlyRα3 expression and p38 pathways, which would be further investigated in the future.

Recently, TXNIP, a known negative regulator of antioxidative protein thioredoxin [65], has emerged to be an attractive target in gouty arthritis [66], cervical inflammation [18], and CNS-related injury or diseases [15–18], implicating TXNIP as an important player in their pathogenesis. In this study, we demonstrate that TXNIP is not only linked to the regulation of neuropathic pain, but also directly regulated by CXCR4 in neuropathic pain. As TXNIP is involved in the regulation of different diseases by controlling their stability at mRNA and protein levels [67], we speculate that the C terminal of CXCR4 protein may stabilize TXNIP by binding to the given three-dimensional structure of TXNIP, which, by preventing the degradation of TXNIP, results in the increase of TXNIP protein in the spinal cord of neuropathic pain mice. It is notable that CXCR4 pulled down by TXNIP is increased by over 3-fold, while the expression of CXCR4 is almost increased by about 1.4-fold in pain models, suggesting there is stronger affinity or binding potency between spinal CXCR4 and TXNIP, at least, in the spinal cord of neuropathic pain mice. Our data provides first direct evidence at molecular and cellular level demonstrating that CXCR4 modulates pain via direct interaction with TXNIP protein. However, which amino acid in TXNIP protein is bound by CXCR4 needs further investigation in the future.

Previous evidence showed that TXNIP linked oxidative stress to the activation of NLRP3 inflammasome [13], which plays a crucial role in innate immunity and inflammation. However, there has been debate on whether nerve injury altered the expression of NLRP3 inflammasome and whether NLRP3 is connected with the process of neuropathic pain. To date, only few studies have examined NLRP3 expression following nerve injury, with conflicting results. In a study by Curto-Reyes et al. [68], spared nerve injury (SNI) induces the development of mechanical allodynia and thermal hyperalgesia but does not change mRNA levels of the NLRP3 inflammasome components, such as ASC, caspase-1, or IL-1β in the spinal cord. The other studies find that CCI-induced neuropathic pain [69], or a brief course of morphine given following CCI-prolonged pain [31], increases the expression of NLRP3 inflammasome protein in the mouse or rat ipsilateral spinal dorsal horn. Additionally, the cellular distribution of NLRP3 in the nervous system is still debatable. NLRP3, in vitro, is expressed in primary cultured rat astrocytes, mouse microglia, and mouse cortical neurons [70–72] but not in cultured primary human neurons, purified microglia, and astrocytes from fetal tissue [73]. Double immunofluorescence staining indicates that NLRP3, in vivo, is mainly expressed in neuron and microglia cells but only few astrocyte cells of the spinal cord of rat [74]. While another recent study shows that lumbar dorsal spinal NLRP3 is co-localized with IBa1 but not NeuN or GFAP [31], in the present study, we demonstrated pSNL caused an increase of NLRP3 protein in the spinal cord of mice. Our results support the model whereby the NLRP3 inflammasome is activated by nerve injury causing amplification of spinal neuroinflammation and neuropathic pain.

NLRP3 activation was affected by multiple cellular proteins or factors. TXNIP binding to NLRP3 is a key signaling mechanism necessary for NLRP3 inflammasome formation and activation; TXNIP inhibition significantly reduced NLRP3, ACS, caspase-1 activity, and IL-1β production [75, 76]. The axis of TXNIP and NLRP3 inflammasome has been confirmed to be an important player in CNS dysfunction or several diseases, such as hippocampus injury [32], type 2 diabetes [13], and brain ischemic stroke [77]. In the present study, we found that nerve injury induced the increase of TXNIP and activated the NLRP3 inflammasome via the formed complex with NLRP3. Therefore, combining the previous finding of specific expression of NLRP3 in spinal microglial cells [31] with our observations of cellular distribution of CXCR4 and TXNIP in spinal microglia and neurons, we speculated that CXCR4 plays an important role in neuropathic pain, at least, partly through microglial TXNIP or the transmitted microglial TXNIP from spinal neurons activating NLRP3 inflammasome in spinal microglial cells. Consequently, TXNIP/NLRP3 inflammasome cascade acts as a supplementary signal pathway in the involvement of CXCR4 in the process of the nociceptive response. The current findings further expand our knowledge about the functional regulatory role of the spinal TXNIP/NLRP3 inflammasome cascade in CNS diseases. However, it would need to be determined whether there is a feedback mechanism of TXNIP regulating the CXCR4 receptor and whether spinal neuronal TXNIP could regulate the microglial NLRP3 via transmission means.

## Conclusion

Our findings demonstrate that downregulation of miR-23a increases spinal CXCR4 expression and subsequently induces neuropathic pain through modulating the TXNIP/NLRP3 inflammasome axis. Notably, upregulation of miR-23a relieves peripheral nerve injury-induced neuropathic pain. These findings reveal a unique mechanism underlying neuropathic pain, which may provide a potential and novel target for the management of neuropathic pain in the future.

## Additional file

Additional file 1: Figure S1. The CXCR4 mRNA expression in spinal cell types. a–c Combined FISH of CXCR4 mRNA (green) and immunofluorescence staining of NeuN (a), GFAP (b), or IBA1 (c) (red) in the ipsilateral spinal cord of normal mice. The antisense probe of CXCR4 mRNA and marker protein antibodies were hybridized to the spinal cord slice of normal mice. Scale bar, 50 μm. Arrows indicate the positive CXCR4 mRNA signal. Figure S2. The TXNIP mRNA expression in spinal cell types. a–c Combined FISH of CXCR4 mRNA (green) and immunofluorescence staining of NeuN (a), GFAP (b), or IBA1 (c) (red) in ipsilateral spinal cord of normal mice. The antisense probe of TXNIP mRNA and marker protein antibodies were hybridized to the spinal cord slice of normal mice. Scale bar, 50 μm. Arrows indicate the positive TXNIP mRNA signal. Figure S3. The knockdown validation of TXNIP siRNA-681 and siRNA-1271 in vivo. a–b TXNIP mRNA (a) and protein (b) change after intrathecal injection of TXNIP siRNA. The scrambled or siRNAs were daily intrathecally injected for 3 consecutive days in normal mice, and spinal cord was harvested at 48 h after last injection, *p* < 0.01. Data are presented as mean ± SEM; ***p* < 0.01 versus Scr group; *n* = 5. (PPTX 2670 kb)

## Abbreviations

3′-UTR: 3′-untranslated region
ASC: Apoptosis-associated speck-like molecule containing CARD domain
CCI: Chronic constriction injury
CNS: Central nervous system
CXCL12: Chemokine C-X-C motif ligand 12
CXCR4: Chemokine CXC receptor 4
DRG: Dorsal root ganglion
FISH: Fluorescence in situ hybridization
IF: Immunofluorescence
Ih: Inhibitor
IL-1β: Interleukin 1β
IP: Immunoprecipitation
miR-23a: MicroRNA-23a-3p
NLRP3: NOD-like receptor protein 3
pSNL: Partial sciatic nerve ligation
PWL: Paw withdrawal latency
PWT: Paw withdrawal threshold
Scr: Scrambled
TXNIP: Thioredoxin-interacting protein
