# Supplementary material for: miRNA-23a/CXCR4 regulates neuropathic pain via directly targeting TXNIP/NLRP3 inflammasome axis
Source: J Neuroinflammation. 2018 Jan 31;15:29. doi: 10.1186/s12974-018-1073-0 (PMC5791181; doi:10.1186/s12974-018-1073-0)
Supplement: Additional file 1: Figure S1. — The CXCR4 mRNA expression in spinal cell types. a–c Combined FISH of CXCR4 mRNA (green) and immunofluorescence staining of NeuN (a), GFAP (b), or IBA1 (c) (red) in the ipsilateral spinal cord of normal mice. The antisense probe of CXCR4 mRNA and marker protein antibodies were hybridized to the spinal cord slice of normal mice. Scale bar, 50 μm. Arrows indicate the positive CXCR4 mRNA signal. Figure S2. The TXNIP mRNA expression in spinal cell types. a–c Combined FISH of CXCR4 mRNA (green) and immunofluorescence staining of NeuN (a), GFAP (b), or IBA1 (c) (red) in ipsilateral spinal cord of normal mice. The antisense probe of TXNIP mRNA and marker protein antibodies were hybridized to the spinal cord slice of normal mice. Scale bar, 50 μm. Arrows indicate the positive TXNIP mRNA signal. Figure S3. The knockdown validation of TXNIP siRNA-681 and siRNA-1271 in vivo. a–b TXNIP mRNA (a) and protein (b) change after intrathecal injection of TXNIP siRNA. The scrambled or siRNAs were daily intrathecally injected for 3 consecutive days in normal mice, and spinal cord was harvested at 48 h after last injection, p < 0.01. Data are presented as mean ± SEM; **p < 0.01 versus Scr group; n = 5. (PPTX 2670 kb) [file 12974_2018_1073_MOESM1_ESM.pptx]

## Slide 1
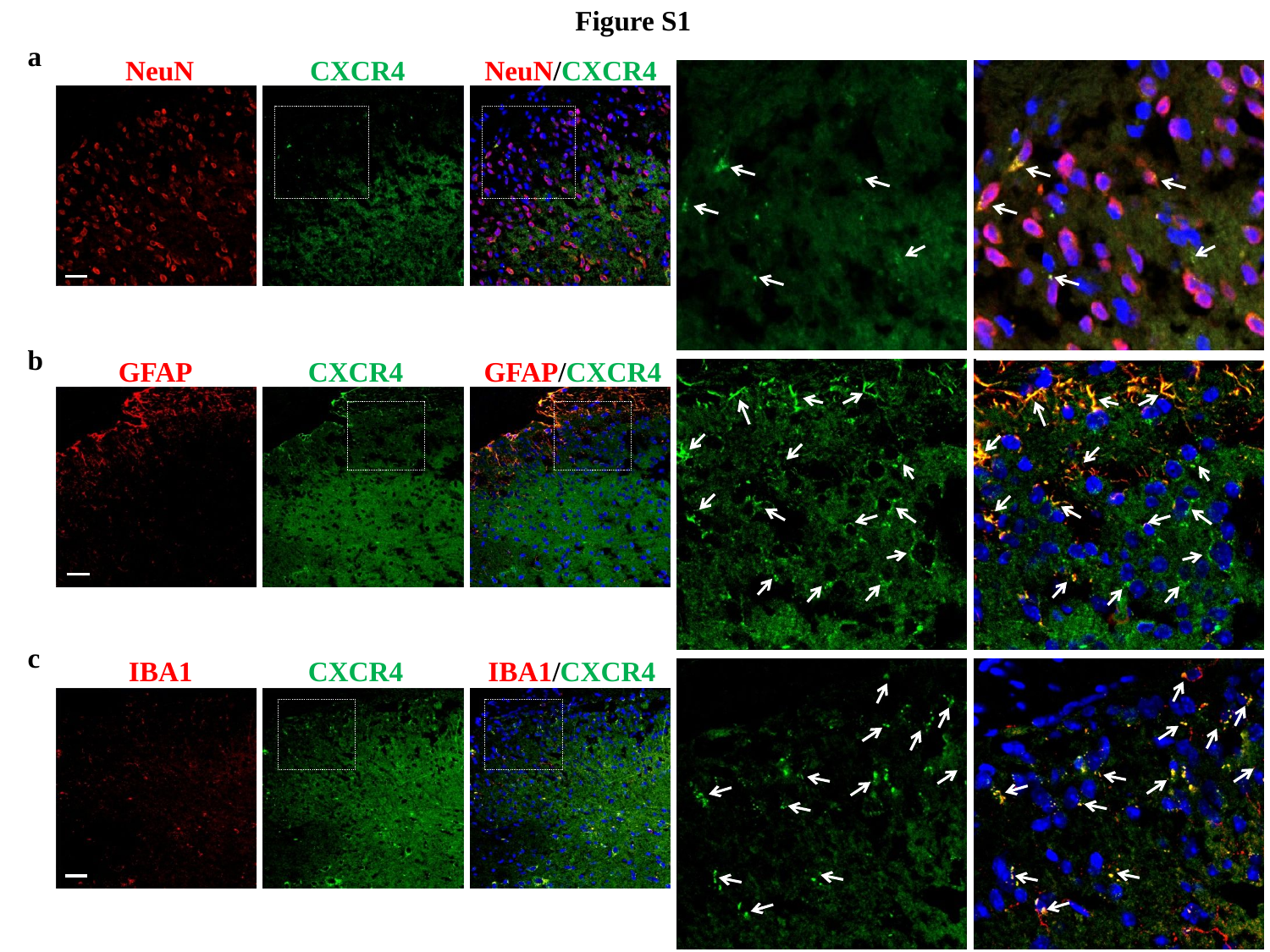

Figure S1
a
NeuN
CXCR4
NeuN/CXCR4
b
GFAP
CXCR4
GFAP/CXCR4
c
IBA1
CXCR4
IBA1/CXCR4

## Slide 2
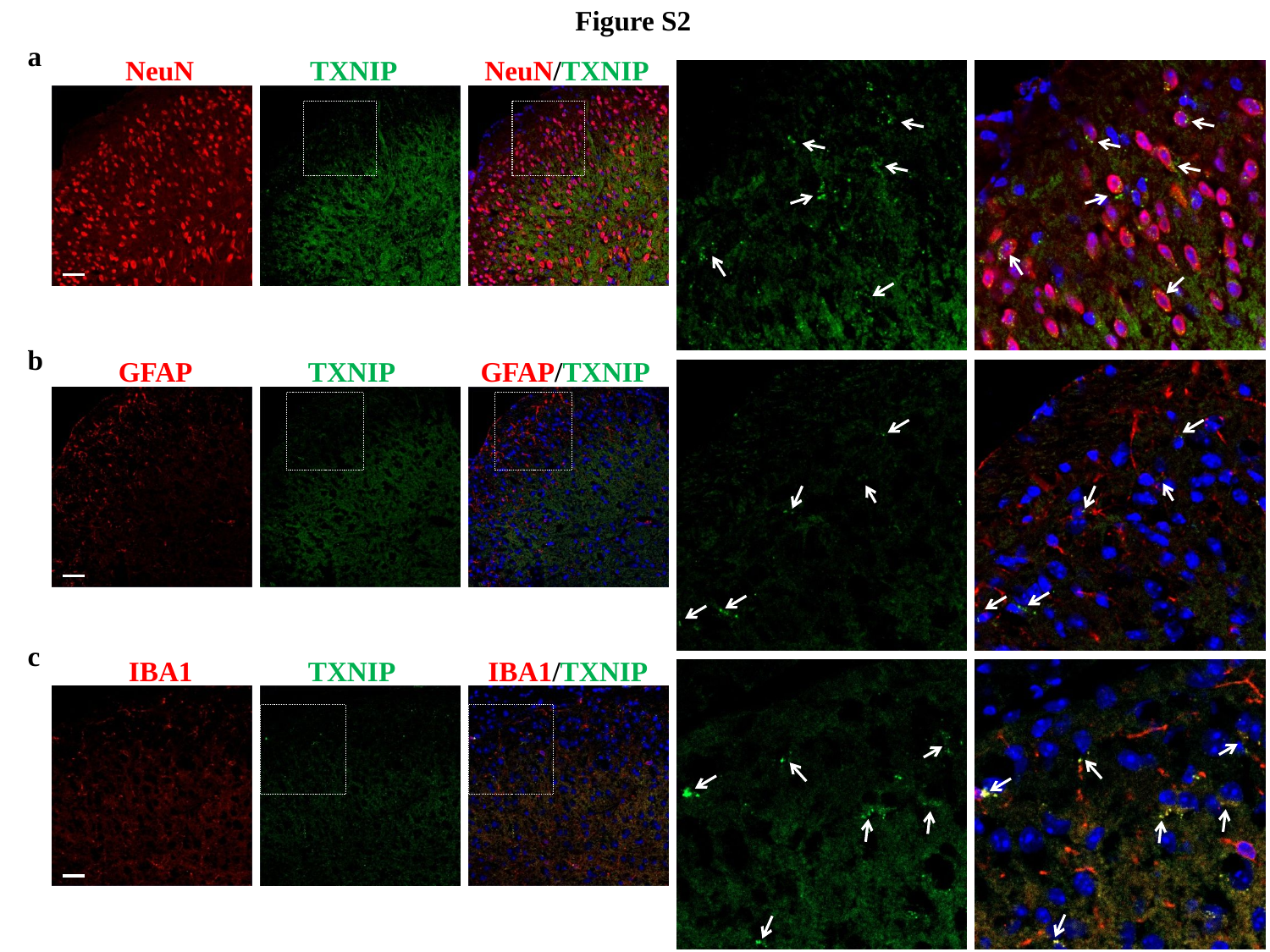

Figure S2
a
NeuN
TXNIP
NeuN/TXNIP
b
GFAP
TXNIP
GFAP/TXNIP
c
IBA1
TXNIP
IBA1/TXNIP

## Slide 3
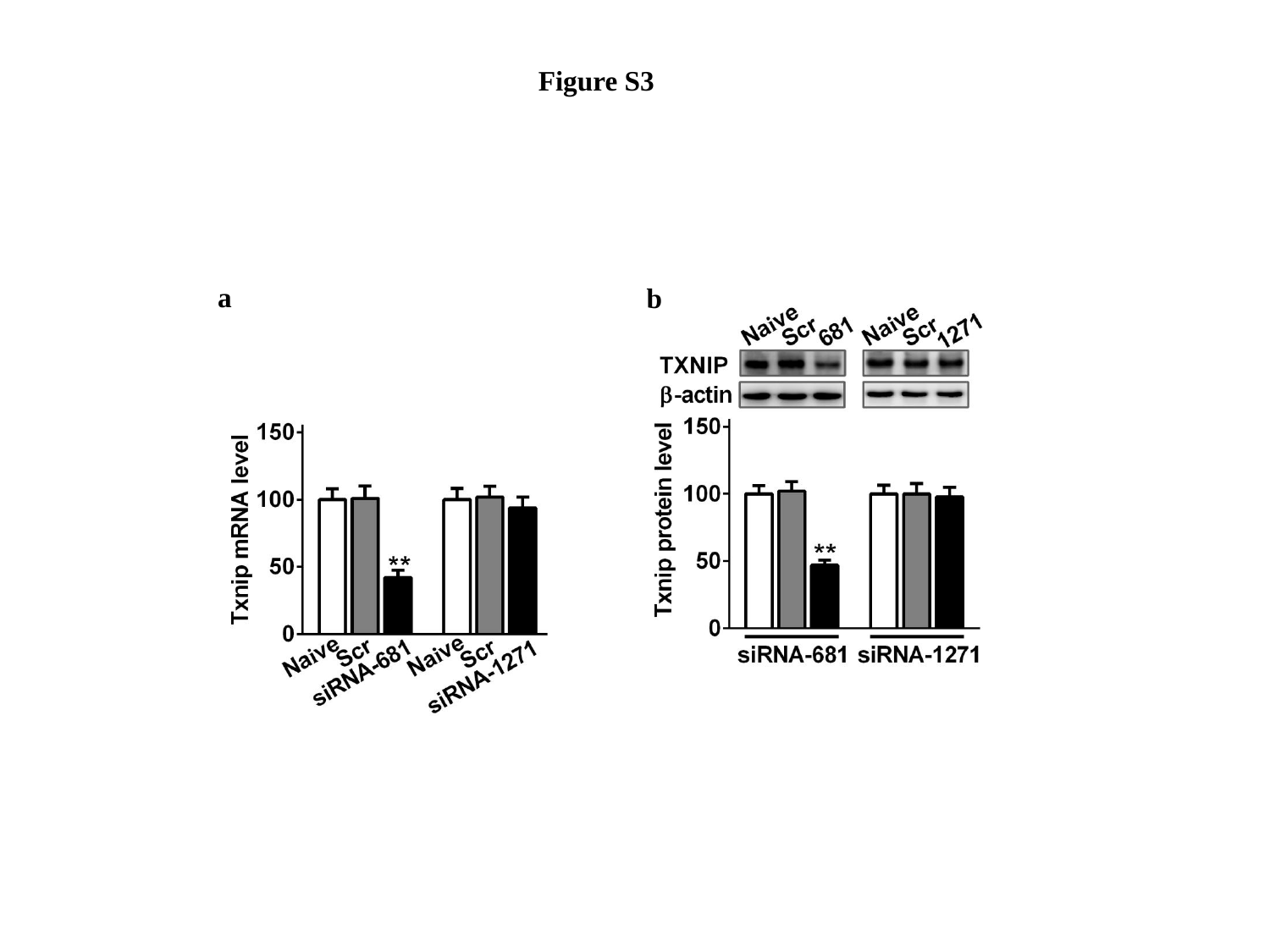

Figure S3
a
b
